# Supplementary material for: Measuring and addressing the childhood tuberculosis reporting gaps in Pakistan: The first ever national inventory study among children
Source: PLoS One. 2019 Dec 30;14(12):e0227186. doi: 10.1371/journal.pone.0227186 (PMC6936771; doi:10.1371/journal.pone.0227186)
Supplement: Attachment — Submitted filename: Reviewer comments_PONE-D-19-14628.pdf [file pone.0227186.s001.pdf]

Review: Measuring and addressing the childhood TB reporting gaps in Pakistan: the first ever national TB inventory study among children

Thank you for the opportunity to review this manuscript.

The authors address a very important topic on childhood TB which is often neglected given the challenges in obtaining sputum in children. While this is an important question, the study approach, analysis and results could be presented better but in some instances are erroneous.

Reviewer's Opinion: Major revision due to the flaws in the analysis – linkage of data which may have introduce bias and due to the erroneous presentation of findings. Please see specific comments below.

Line 70: "According to TB notification 2017 in Pakistan, only 10% of cases were children." How does this compare with what is expected in the population based on the WHO estimates for TB burden?

Line 71: "In Pakistan, the private health sector has grown rapidly, and TB drugs are available in

private pharmacies." - are these free

line 72: Refrain from starting a sentence with a number(s)

line 76: statement is not clear- refers to private sector then public-private relationships...there is need for clarity on the approach

Additional details are required in the background to provide more context and allude to what resources are available

What guidelines exist for TB diagnosis in children and how is TB diagnosis made in children in Pakistan – you could provide a figure of the algorithm

Line 90 - How many districts and health facilities were available in total (sampling frame)

line 101, Sample size ---given the proposed percentage of 27% under-reporting, estimating a sample size that allows 25% margin of error (translates to between 4000-27500

Line 132 - MTB/RIF - write in full

Line 179 -183 "Record linkage was carried out as outlined in guidance issued by WHO [6], by cross-checking the notifications of non-NTP facilities, compared with official public district TB

registers, using the combination of the first name, father's name and family name in English182 language as identifiers".....the number of fields used for matching/record linkage is so small and uses variables which are not likely to result to unique identifiers and might have contribute to mis-classification

How successful was the matching and record linkage?

Line 203: "The level of underreporting nationally was 78% with marked differences between provinces." As mentioned above, this might be as a result of poor linkage rather than under reporting. As above, more data is required on record linkage

Also, useful to provide a table with quality of documentation for variables of interest and provide a comparison between public and private sector…it is possible there is better linkage in the private due to better quality of data and hence biasing the results

Table 1 is difficult to understand- the authors present both row and column percentages

Table 3: This is a little confusing …My understanding is that the TB inventory was the gold standard for comparison and therefore under should be defined as ((b+c)-a)/ (b+c). In the current approach you are double counting those reported in NTP in the denominator – some of those in 'a' are also in 'b'

Provide an additional table with some of the characteristics of the selected facilities/districts – how many were tertiary; public vs private; capacity for TB diagnosis etc, what is the estimated disease burden in the districts

The authors should provide reasons why the data are not publicly available

Discussion:

Better description of context on the possible reasons for the high diagnosis rate in the private sector vs public sectors. Is it because there is a difference in utilization and access for other paediatric services? Could the low reporting rates be linked to poor availability of resources to support TB diagnosis in the public sector?

Line 268: "Currently, child TB care services are confined to only tertiary care hospitals in Pakistan;" If this is true, how many tertiary hospitals were included in the study, this might explain the massive under reporting (data not captured) in the public sector….
