# Supplementary material for: Measuring and addressing the childhood tuberculosis reporting gaps in Pakistan: The first ever national inventory study among children
Source: PLoS One. 2019 Dec 30;14(12):e0227186. doi: 10.1371/journal.pone.0227186 (PMC6936771; doi:10.1371/journal.pone.0227186)
Supplement: Attachment — Submitted filename: Editors summary of suggested revisions for manuscript PONE-D-19-14628_17July2019.pdf [file pone.0227186.s002.pdf]

Editor's summary of suggested reviewer revisions for manuscript PONE-D-19-14628 -- Measuring and addressing the childhood TB reporting gaps in Pakistan: The First Ever National TB Inventory

General comments

"Major revision due to the flaws in the analysis – linkage of data which may have introduce bias and due to the erroneous presentation of findings."

"The article focuses on underreporting of childhood tuberculosis, which is the urgent problem and should be emphasized as a publication in PLOSONE. This could be an important step to improve care in childhood TB in Pakistan in the future. However, some points should be concerned."

"These investigators have documented significant underreporting of childhood TB cases in Pakistan. A prospective cross-sectional study design was used. Districts were utilized as the unit, with sampling done within each district. Study design was appropriate; all sectors potentially diagnosing childhood TB cases were instructed to complete forms with demographic and diagnostic information for all childhood TB diagnosed within the prospective study timeframe. They were instructed to not otherwise change any practices. Data from these prospective case reports were then compared to the National database for the same time period, with appropriate assumptions made for case linking between the two databases. Results demonstrate significant under-reporting of cases – nearly 80%. Males were more likely under-reported than females. Surprisingly bacteriologically diagnosed cases were under-reported at the same percentage as clinical diagnosis."

Major Revisions

Study design and analysis considerations (Methods and Results sections):

- Methodology: please give study design name

Sampling--

- Line 90 - How many districts and health facilities were available in total (sampling frame)
- Line 101, Sample size ---given the proposed percentage of 27% under-reporting, estimating a sample size that allows 25% margin of error (translates to between 4000-27500)
- In 2017, Pakistan is a high burden TB country with estimated incidence (includes HIV+TB) of 525,000 cases. It is generally reported that at least 10%–15% of cases in the world and up to 25% of those arising in countries with high TB burden occur in children. The authors showed 5,258 childhood TB cases from the districts that sampled in a 3-month-period study. How many total childhood TB cases in the Pakistan reflected from this number?
- How to select the 12 districts across the country? In page 5, "with probability proportional to population size provinces and regions" (Table 1)?? No detail of this in Table 1.
- Kindly mention the eligibility criteria for selection of study subjects
- It is suggested to use "Sample selection" instead of sampling design.

Record linkage --

- Line 179 -183 "Record linkage was carried out as outlined in guidance issued by WHO [6], by cross-checking the notifications of non-NTP facilities, compared with official public district TB registers, using the combination of the first name, father's name and family name in English182 language as identifiers".....the number of fields used for matching/record linkage is so small and uses variables which are not likely to result to unique identifiers and might have contribute to mis-classification How successful was the matching and record linkage?

- Line 203: “The level of underreporting nationally was 78% with marked differences between provinces.” As mentioned above, this might be as a result of poor linkage rather than under reporting. As above, more data is required on record linkage

Presentation of results (Tables and Figures):

- Also, useful to provide a table with quality of documentation for variables of interest and provide a comparison between public and private sector…it is possible there is better linkage in the private due to better quality of data and hence biasing the results
- Table 1 is difficult to understand- the authors present both row and column percentages
- How to select the 12 districts across the country? In page 5, “with probability proportional to population size provinces and regions” (Table 1)?? No detail of this in Table 1.
- Table 3: This is a little confusing …My understanding is that the TB inventory was the gold standard for comparison and therefore under should be defined as ((b+c)-a)/ (b+c). In the current approach you are double counting those reported in NTP in the denominator – some of those in ‘a’ are also in ‘b’
- The Venn diagram needs to be better explained. I assume that the overlaps are where a case appeared in both databases. However, the way it is labeled and the legend reads, it appears that the overlap are cases whose TB was diagnosed in both facilities. Please reword the legend to clarify.
- 3. The authors might consider use of a map utilizing the data in Table 3. The map could also illustrate where the non-surveyed tribal areas are located.
- In the manuscript, table 1 is "Characteristic of children" please make corrections

Discussion

- Better description of context on the possible reasons for the high diagnosis rate in the private sector vs public sectors. Is it because there is a difference in utilization and access for other paediatric services? Could the low reporting rates be linked to poor availability of resources to support TB diagnosis in the public sector?
- Line 268: “Currently, child TB care services are confined to only tertiary care hospitals in Pakistan;” If this is true, how many tertiary hospitals were included in the study, this might explain the massive under reporting (data not captured) in the public sector….

Availability of data:

- The authors should provide reasons why the data are not publicly available

Minor Revisions

Introduction:

- Line 70: “According to TB notification 2017 in Pakistan, only 10% of cases were children.” How does this compare with what is expected in the population based on the WHO estimates for TB burden?
- Line 71: "In Pakistan, the private health sector has grown rapidly, and TB drugs are available in private pharmacies." - are these free
- Introduction section line 82, please give reference

Study design and analysis considerations:

- Data quality audit – For data quality audit which of the records is taken as correct or gold standard?

Copy editing considerations:

- line 72: Refrain from starting a sentence with a number(s)
- Line 132 - MTB/RIF - write in full
- Full title is not aligned with short title, kindly make corrections
- Abstract should start with an "Introduction"
- Kindly rephrase following lines in the abstract" A nationwide, prospective cross sectional, cluster-sampled design was used" this is not a study design
- Do not use abbreviations (e.g., TB) in the title.
- In general, always spell out the full name of abbreviations or acronyms the first time used in the text.
- Prior to re-submission, entire paper would benefit from, and be strengthened by, external review by someone whom possesses excellent proficiency in English grammar, to ensure accuracy and clarity. For example, in addition to other suggestions provided by reviewers: line 69; line 156; lines 252 – 262; line 269; line 277.

Discussion:

- Underreporting was 78%; approximately 90% of TB cases are from non-NTP private health facilities. Should we discuss more why the NTP and public health facilities do not work?
- The tests used for TB diagnosis is an important issue. The molecular test, such as GeneXpert is available and used countrywide or only in some cities. Of note, in Chiniot and Karachi district has high percentage of the cases that bacteriologically confirmed which is different from other districts even in the same provinces. Any explanation for this? Surprisingly, almost 30% in Chiniot was bacteriologically confirmed but very low reporting (90% in Table 3). So this is not the problem of uncertain TB diagnosis. The main reason of this underreporting should be also discussed.
- Importantly, the authors should comment more on policies of the National Tuberculosis Control Program in Pakistan as well including routine reporting of childhood TB, BCG vaccine at birth, treatment of latent TB infection in household contact children, support of GeneXpert and reimbursement, that may bring better understanding to the reader.
- The discussion is appropriate but could be improved by suggestions to make reporting more robust. For instance could it be made mandatory to report a case when a chemist receives a prescription for treatment drugs?
- Brief discussion regarding thoughts about the gender differences detected, in terms of TB diagnosis and reporting, would strengthen the paper.
- it would be nice to discuss reasons for gaps in reporting or underreporting
- in discussion section please give some information on 'periphery'

Study challenges and limitations:

- The authors might want to comment on the possibility of a Hawthorne effect, where during the study facilities were more likely to report than usual. This might especially have occurred if the participants were aware of the study purpose.

Discretionary Revisions

- line 76: statement is not clear- refers to private sector then public-private relationships...there is need for clarity on the approach

- Additional details are required in the background to provide more context and allude to what resources are available What guidelines exist for TB diagnosis in children and how is TB diagnosis made in children in Pakistan – you could provide a figure of the algorithm
- It would be interesting if details of "data collection through cell phone" is shared such as name of android device, name of android application" etc...
- Provide an additional table with some of the characteristics of the selected facilities/districts – how many were tertiary; public vs private; capacity for TB diagnosis etc, what is the estimated disease burden in the districts
- Authors may want to consider having a separate sub-section labeled “Challenges” or “Limitations.”
