# Supplementary material for: Measuring and addressing the childhood tuberculosis reporting gaps in Pakistan: The first ever national inventory study among children
Source: PLoS One. 2019 Dec 30;14(12):e0227186. doi: 10.1371/journal.pone.0227186 (PMC6936771; doi:10.1371/journal.pone.0227186)
Supplement: Attachment — Submitted filename: Rebuttal letter.docx [file pone.0227186.s003.docx]

Highlighted comments are incorporated in revised manuscript as suggested. The reviewers comments are answered below

1..Thank you for providing the following Data Availability Statement: "Data can be accessed through “drrazia@ntp.gov.pk” or “aashifa.yaqoob@ntp.gov.pk” by contacting IRC ethics committee, Common management unit (HIV/AIDS, TB & Malaria) to which data requests can be sent."

In addition, thank you for also providing the following information in your cover letter: "some data sharing restrictions are imposed by the program. Special permission for program is required for data availability request because of office policy restriction for data sharing. Data can be accessed through “drrazia@ntp.gov.pk” or “aashifa.yaqoob@ntp.gov.pk” by contacting IRC ethics committee, Common management unit (HIV/AIDS, TB & Malaria) to which data requests can be sent."

Before we proceed, can you please provide a non author point of contact for the IRC ethics committee (such as an email). Please note, it is not acceptable for the author to be the sole named individual responsible for ensuring data access.

In addition, we would like to propose the following Data Availability statement for your approval:

"Some data sharing restrictions are imposed by the program. Special permission for program is required for data availability request because of office policy restriction for data sharing. Data Can be accessed by contacting IRC Ethics Committee [CONTACT INFORMATION YOU PROVIDE], Common Management unit (HIV/AIDS, TB & Malaria) to which data requests can be sent."

Please confirm the statement above is complete and correct. Once you have approved this statement and provided the contact information, we will update your data availability statement on your behalf.

Reply: Contact information is updated as suggested. Line # 199-206

*** we note the following request is still outstanding from your decision letter: ***

2..In ethics statement in the manuscript and in the online submission form, please provide additional information about the patient records used in your study. Specifically, please ensure that you have discussed whether all data were fully anonymized before you accessed them and/or whether the IRB or ethics committee waived the requirement for informed consent.

Reply: Ethical statement is revised as per highlighted concern. Line # 332-335
